# Incidence and risk factors of adjacent segment disease following posterior decompression and instrumented fusion for degenerative lumbar disorders

**DOI:** 10.1097/MD.0000000000006032

**Published:** 2017-02-03

**Authors:** Hui Wang, Lei Ma, Dalong Yang, Tao Wang, Sen Liu, Sidong Yang, Wenyuan Ding

**Affiliations:** Department of Spine Surgery, The Third Hospital of HeBei Medical University, Shijiazhuang, China.

**Keywords:** adjacent segment disease, degenerative lumbar disorders, posterior decompression and instrumented fusion

## Abstract

The purpose of this study was to explore incidence and risk factors of adjacent segment disease (ASD) following posterior decompression and instrumented fusion for degenerative lumbar disorders, and hope to provide references in decision making and surgical planning for both spinal surgeon and surgically treated patients.

By retrieving the medical records from January 2011 to December 2013 in our hospital, 237 patients were retrospectively reviewed. According to the occurrence of ASD at follow up, patients were divided into 2 groups: ASD and N-ASD group. To investigate risk values for the occurrence of ASD, 3 categorized factors were analyzed statistically: *Patient characteristics*: age, sex, body mass index (BMI), bone mineral density (BMD), duration. Surgical variables: surgical strategy, number of fusion level, surgery segment, surgery time, blood loss, intraoperative superior facet joint violation. *Radiographic parameters*: preoperative lumbar lordosis, preoperative angular motion at adjacent segment, preoperative adjacent segment disc degeneration, preoperative paraspinal muscle degeneration.

Postoperative ASD was developed in 15 of 237 patients (6.3%) at final follow up. There was no statistically significant difference between the 2 groups in patient characteristics of age, sex composition, BMD, duration, while the BMI was higher in ASD group than that in N-ASD group. There was no difference in surgical variables of surgical strategy, number of fusion level, surgery segment, surgery time, blood loss, while intraoperative superior facet joint violation was more common in ASD group than that in N-ASD group. There was no difference in radiographic parameters of preoperative lumbar lordosis, preoperative paraspinal muscle degeneration, while preoperative adjacent segment disc degeneration were more severe in ASD group than that in N-ASD group. The Logistic regression analysis revealed that, BMI >25 kg/m^2^, preoperative disc degeneration, and superior facet joint violation were independently associated with ASD.

In conclusion, higher BMI, preoperative disc degeneration at adjacent segment and intraoperative superior facet joint violation are risk factors for ASD. Patients who are overweight or obesity and with preoperative disc degeneration at adjacent segment should be fully informed the risk of ASD. For surgeons, it is essential to prevent superior facet joint violation in pedicle screw insertion procedure.

## 1 Introduction

Spinal fusion has become a commonly performed procedure in recent decades for treating degenerative lumbar disorders, and is supposed to eliminate abnormal motion and instability at the symptomatic degenerated levels.^[1–3]^ Although a large number of studies have proved the effectiveness and reliability of the procedure, complications related to fusion cannot be underestimated.^[4–7]^ There are convincing biomechanical and clinical data that spinal fusion creates a significant compensatory increase in the motion of the adjacent segment as a result of the increased rigidity of the fused segment.^[8]^ The development of adjacent segment degeneration or adjacent segment disease (ASD) is considered to be potential long-term complications of spinal fusion, the former represents radiographic change in discs adjacent to the surgically treated levels, whereas the latter is defined as the pathologic process associated with disc degeneration, leading to deterioration of the surgical outcome and sometimes requiring further surgical treatment.^[9]^

In the previous literature, the risk factors for the occurrence of adjacent segment degeneration following spinal fusion have been studied extensively and deeply, including older age, female, expression of the estrogen receptor, the number of instrumented level, preexisting degenerative condition at an adjacent motion segment, sagittal alignment change, etc.^[10–16]^ To the best of our knowledge, little study focusing on the risk factors of ASD after spinal fusion surgery. The purpose of this study is therefore to explore incidence and risk factors of ASD following posterior decompression and instrumented fusion for degenerative lumbar disorders, and hope to provide references in decision making and surgical planning for both spinal surgeons and surgically treated patients.

## 2 Materials and methods

### 2.1 Subjects

This is a retrospective study, it was approved by the Institutional Review Board of the Third Hospital of HeBei Medical University before data collection and analysis. The inclusion criteria: lower degenerative lumbar disorders including lumbar disc herniation, lumbar spinal stenosis, degenerative lumbar spondylolisthesis (Taillard index <30%). Surgical strategy including posterior lumbar interbody fusion (PLIF) and transforaminal lumbar interbody fusion (TLIF). Follow-up duration more than 2 years with complete radiological data including lumbar anteroposterior (A/P) and lateral X-ray at preoperation, early postoperation, and final follow-up, computed tomography (CT) or magnetic resonance imaging (MRI) at preoperation and final follow-up. The exclusion criteria: Patients treated for non degenerative disorders, such as trauma, tumor, infection, inflammation, or isthmic spondylolisthesis. Patients treated with anterior or lateral lumbar fusion surgery, minimally invasive lumbar fusion surgery.

By retrieving the medical records from January 2011 to December 2013 in our hospital, 237 patients met both the inclusion and exclusion criteria were retrospectively reviewed. One hundred thirty-one females and 106 males with mean age of 53.2 ± 10.8 years (range from 37 to 69 years). There were 88 cases of disc herniation, 84 cases of spinal stenosis, 65 cases of spondylolisthesis. Ninety-eight cases undertook TLIF (75 of them received 1-level TLIF and 23 patients received 2-level TLIF) and 139 cases undertook PLIF (103 of them received 1-level PLIF and 36 patients received 2-level PLIF).

### 2.2 Radiological and clinical evaluation

**Figure 1:**
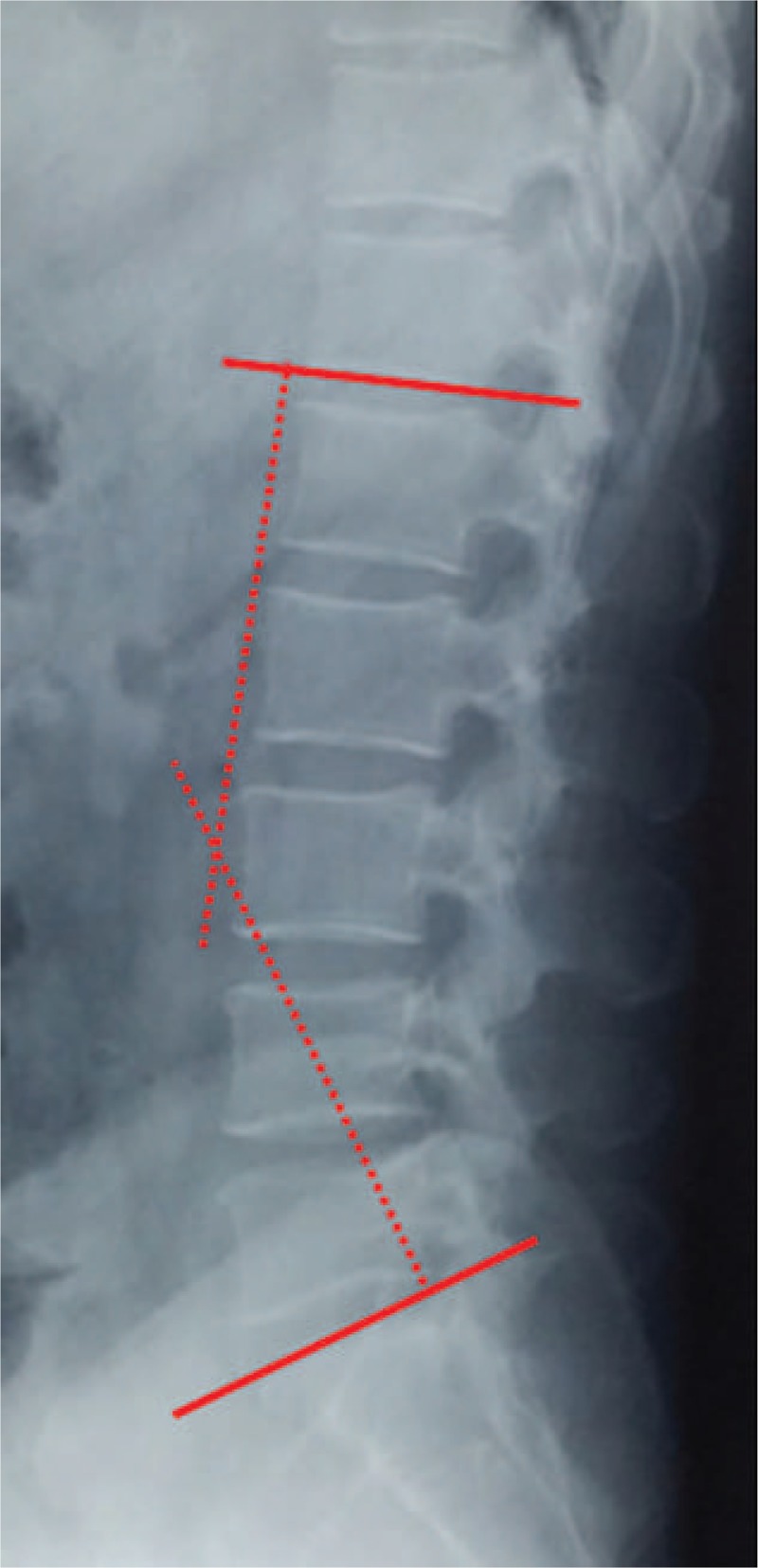
Lumbar lordosis was measured from T12 inferior endplate to S1 superior endplate by the Cobb method.

**Figure 2:**
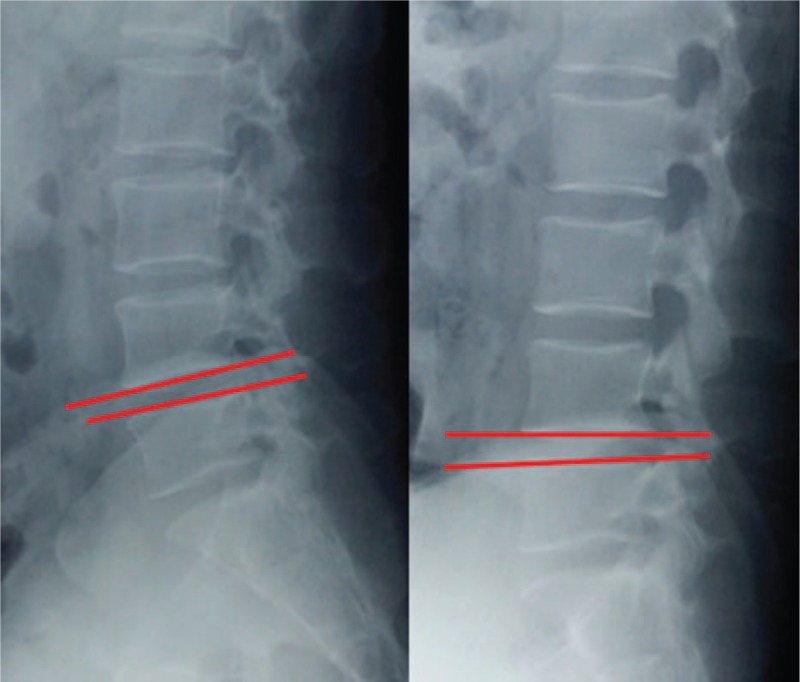
Angular motion was measured between the inferior end plate line of the upper vertebral body and superior end plate line of the lower vertebral body on flexion and extension lateral radiographs.

**Figure 3:**
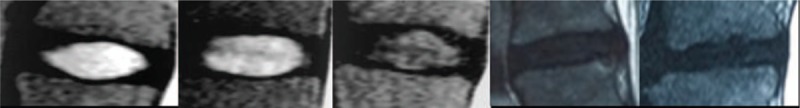
Pfirrmann Grade I: the structure of the disc is homogeneous, with bright hyperintense white signal intensity any normal disc height. Grade II: the structure of the disc is inhomogeneous, with the hyperintense white signal. Grade III: the structure of the disc is inhomogeneous, with an intermittent gray signal intensity. Grade IV: the structure of the disc is inhomogeneous, with a hypointense dark gray signal intensity. Grade V: the structure of the disc is inhomogeneous, with a hypointense black signal intensity.

**Figure 4:**
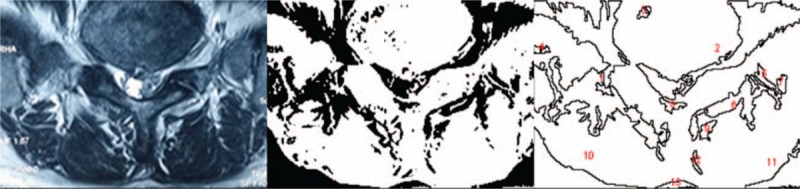
Fatty infiltration rate (FIR) of paraspinal muscles (multifidus and erector spinae) was calculated by subtracting the muscle without the fat value from the total muscle value. Left is the original image, middle is the image of fat left, and right is the calculation of fat area by software.

Lumbar lordosis (LL) was measured from T12 inferior endplate to S1 superior endplate by the Cobb method on lateral X-ray (Fig. 1). Angular motion at the adjacent segment was measured between the inferior endplate line of the upper vertebral body and superior endplate line of the lower vertebral body on flexion and extension lateral radiographs (Fig. 2). Data measurements were performed 3 times with 200% magnification for accuracy by the first and second authors independently, and the mean value was used for analysis. Disc degeneration on MRI was rated from grade 1 to 5 by using the classification system of Pfirrmann et al^[17]^ (Fig. 3). Fatty infiltration rate (FIR) of paraspinal muscles (multifidus and erector spinae) was calculated by subtracting the muscle without the fat value from the total muscle value, and the images were adjusted with the image processing software (Image J, version 1.48, USA) (Fig. 4).

Adjacent segments disease was defined as the pathologic process associated with disc degeneration leading to clinical symptoms, such as radiculopathy, stenosis, and instability.^[18]^ According to the occurrence of ASD at follow up, patients were divided into 2 groups: ASD group and N-ASD group. To investigate risk values for the occurrence of ASD, 3 categorized factors were analyzed statistically: Patient characteristics: preoperative data of age, sex, body mass index (BMI), bone mineral density (BMD), the duration of disease (from first symptoms to operation). *Surgical variables*: surgical strategy (TLIF vs PLIF), number of fusion level (1 level vs 2 level), surgery segment (L_4–5_, L_5_–S_1_, L_4_–S_1_), surgery time, blood loss, intraoperative superior facet joint violation. *Radiographic parameters*: preoperative lumbar lordosis (LL), preoperative angular motion at adjacent segment, preoperative adjacent segment disc degeneration (Pfirrmann grade), preoperative paraspinal muscle degeneration (FIR).

### 2.3 Statistical analysis

Data were analyzed using Statistical Product and Service Solutions software (version 17; SPSS, Chicago, IL). Continuous variables were measured as mean ± standard deviation, and categorical variables were expressed as frequency or percentages. An independent *t* test was used to analyze the difference of continuous variables between 2 groups. An *χ*^2^ analysis and Fisher exact test were used to examine the differences among categorical variables. Logistic regression analysis was used to analyze the assumed risk factors with backward elimination, in which variables with a significance level of >0.10 were removed. The confidence interval of the odds ratio (OR) was 95%.

## 3 Results

Postoperative ASD was developed in 15 of 237 patients (6.3%) at follow up, all of them presented ASD above the surgery segment, and were enrolled as ASD group. The mean follow up duration was 2.6 ± 0.2 years in ASD group and 2.5 ± 0.3 years in N-ASD group, presenting no significant difference (*P* = 0.691).

There was no statistically significant difference between the 2 groups in patient characteristics of age, sex composition, BMD, duration, while the BMI was higher in ASD group than that in N-ASD group (Table 1). There was no difference in surgical variables of surgical strategy, number of fusion level, surgery segment, surgery time, blood loss, while intraoperative superior facet joint violation was more common in ASD group than that in N-ASD group (Table 2). There was no difference in radiographic parameters of preoperative lumbar lordosis, preoperative paraspinal muscle degeneration, while preoperative adjacent segment disc degeneration were more severe in ASD group than that in N-ASD group (Table 3).

**Table 1:**
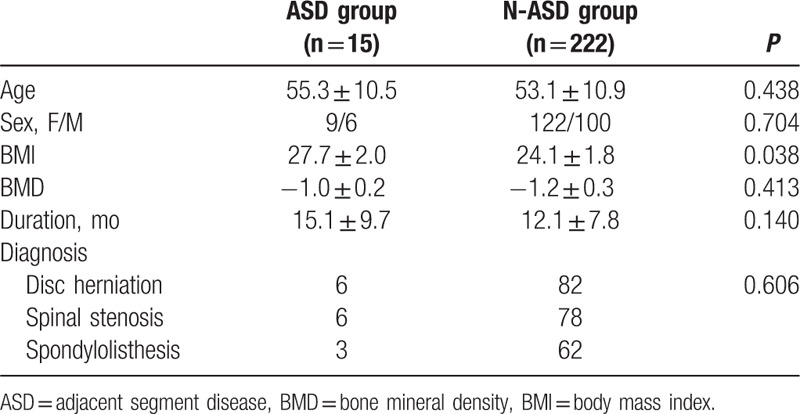
Comparison of patient characteristics between ASD group and N-ASD group.

**Table 2:**
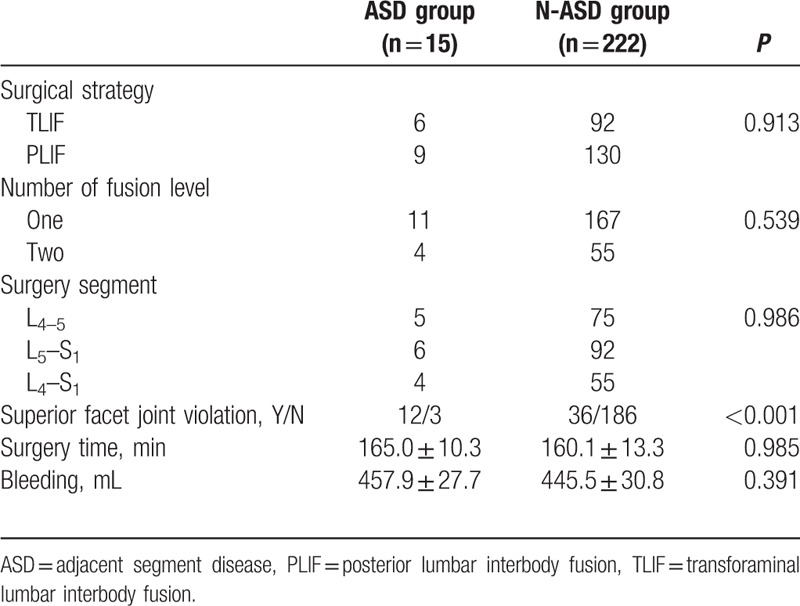
Comparison of surgical variables between ASD group and N-ASD group.

**Table 3:**
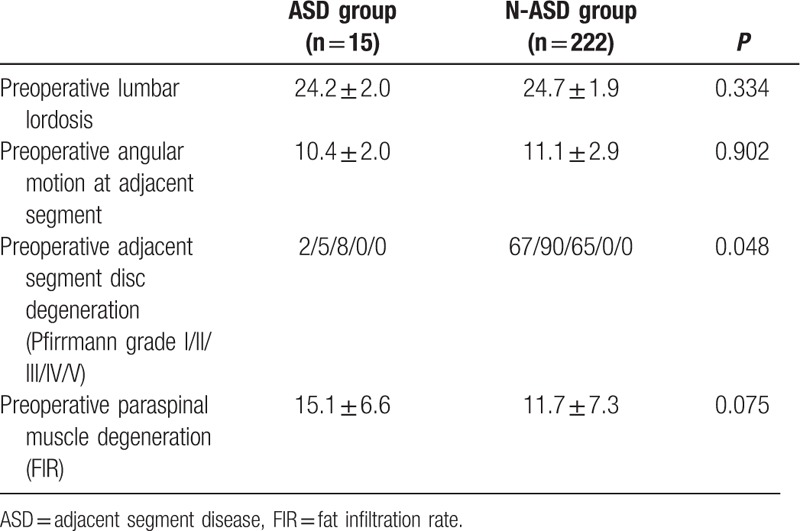
Comparison of radiographic parameters between ASD group and N-ASD group.

The following variables were entered into the logistic regression model: age, sex, BMI, BMD, duration, surgical strategy, number of fusion level, surgery segment, surgery time, blood loss, intraoperative superior facet joint violation, preoperative lumbar lordosis, preoperative adjacent segment disc degeneration, preoperative paraspinal muscle degeneration. The logistic regression analysis revealed that, BMI >25 kg/m^2^, preoperative disc degeneration, and superior facet joint violation were independently associated with ASD (Table 4).

**Table 4:**
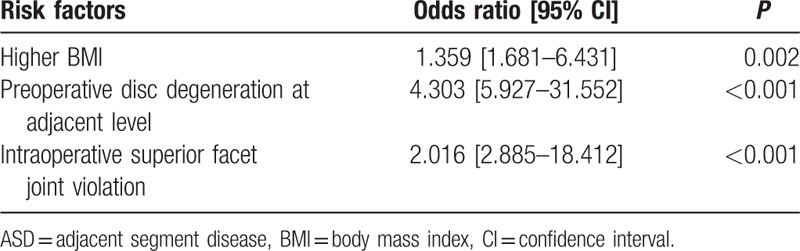
Risk factors for ASD, identified by logistic regression analysis.

## 4 Discussion

In the present study, 6.3% of the patients experienced postoperative ASD, proximal adjacent segment is more involved to be seen than the distal adjacent segment, which is consistent with previous clinical and biomechanical studies.^[10,19]^ Among the risk factors, higher BMI and preoperative disc degeneration at adjacent level were significantly and independently associated with the occurrence of ASD, and can be assessed before surgery. Moreover, intraoperative superior facet joint violation was also a risk factor; these variables were not confounded by other variables that potentially affect postoperative ASD.

BMI is an objective and simple indicator and is accepted universally, as the World Health Organization defines overweight and obesity as BMI values more than 25 and 30 kg/m^2^, respectively.^[20]^ Symmons et al^[21]^ studied women with age range of 45 to 64 years and mean follow-up duration of 9 years, and demonstrated that increased BMI was a risk factor of disc degeneration. Liuke et al^[22]^ also provided evidence that BMI more than 25 kg/m^2^ increases the risk of lumbar disc degeneration. In the present study, BMI value more than 25 kg/m^2^ was found to be a risk factor for the postoperative ASD in patients undergoing posterior decompression and instrumented fusion for degenerative lumbar disorders, which is consistent with the previous study by Ou et al.^[23]^ Increased loading of the spine causes the intervertebral disc to lose height and less ability to absorb a force, leading to abnormal loading on surrounding facet joints, spinal ligaments, and paraspinal muscles.^[24,25]^ Moreover, the paraspinal muscle strength in overweight or obesity patients is not so good comparing to the healthy weight, but it is necessary to strip the muscles from the spinous process and laminae in operation exposure, traction of the paraspinal muscles is also inevitable in the procedure of decompression and instrumented fusion, which may decrease the muscle function postoperatively. If the paraspinal muscles cannot afford enough strength to maintain upright posture, it may accelerate the degeneration of intervertebral disc and articular process, especially in the segment above the fusion level.^[26]^ Therefore, BMI more than 25 kg/m^2^ not only may be a risk factor related to the natural degeneration of healthy spines, but also may play an important part in ASD. On the basis of the result mentioned above, we supposed that controlling body weight before and after surgery could provide opportunities to reduce the incidence of ASD, improve therapeutic outcome and patients’ satisfaction.^[27]^

**Figure 5:**
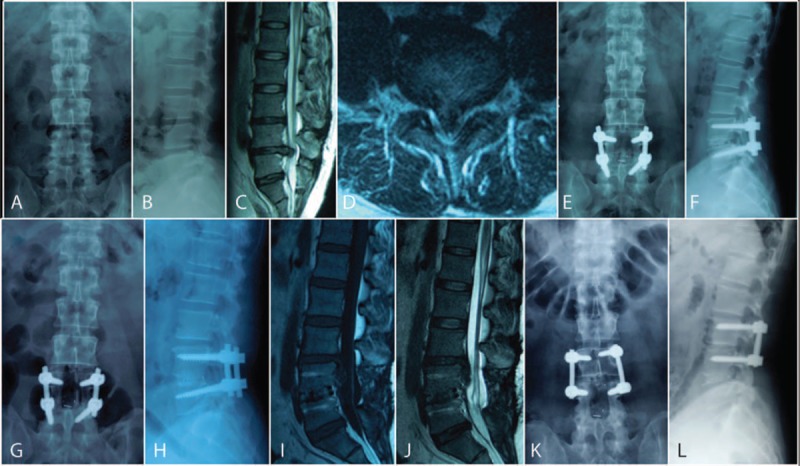
(A–D) Preoperative L_4–5_ disc herniation, and L_3–4_ disc degeneration of Pfirrmann Grade III on MRI. (E and F) L_4–5_ instrumented fusion without intervention to the L_3–4_ disc. (G and H) Bony fusion between cage and endplate in L_4–5_ disc space at 1 year follow up. (I and J) Intervertebral disc prolapse on L_3–4_ 27 months after spinal fusion of L_4–5_. (K and L) Revision surgery of L_5_ pedicle screws taken off and L_3–4_ instrumented fusion was performed.

Postoperative adjacent segment degeneration developed more frequently in patients who had advanced disc degeneration preoperatively, it has been confirmed by both clinical case study and biomechanical analysis.^[19,28–30]^ Anandjiwala et al^[19]^ prospectively reviewed 74 consecutive patients who underwent instrumented lumbar/lumbosacral fusion with a minimum follow-up of 5 years, and demonstrated that patients with preoperative disc degeneration at an adjacent level were more at risk for the development of adjacent segment degeneration. In the present study, we confirm that preoperative disc degeneration at adjacent level was a significant risk factor for postoperative ASD (Fig. 5). Our finding is partly consistent with the studies mentioned above, for adjacent segment degeneration and ASD are not the same entity. The former is a radiological finding, without any clinical problem; while the latter is always present clinical symptoms, revision surgery is required in some cases. Instrumented fusion results in decreased elasticity and increased stiffness of lumbar segment, which leads to biomechanical changes at the adjacent motion segment including stress concentration, increases in segmental hypermobility and intradiscal pressures.^[31]^ For healthy free mobile segment adjacent to lumbar fusion, these biomechanical alterations certainly could contribute to the progressive degeneration postoperatively.^[32]^ While for the degenerated lumbar intervertebral disc adjacent to the fusion segment, the inherent decreased function and the biomechanical alterations make it more vulnerable to experience degeneration after instrumented fusion surgery.^[16]^ There is a controversy in the selection of upper fusion segment when a patient already has a comparable disc degeneration at the adjacent segment to target fusion level. If the adjacent segment is not included in the extent of fusion, it might aggravate the adjacent disc degeneration due to the vulnerability of the adjacent disc. If the adjacent segment is included in the extent of fusion, it consequently would lead to lengthening of the fusion, thereby resulting in an increased potential of developing new adjacent segment degeneration.^[16,33]^ Therefore, no matter the fusion extent to be planned, patients with preoperative disc degeneration adjacent to the fusion segment should be well informed the risk of ASD before surgery, and should not be excluded from the benefit of fusion surgery.

**Figure 6:**
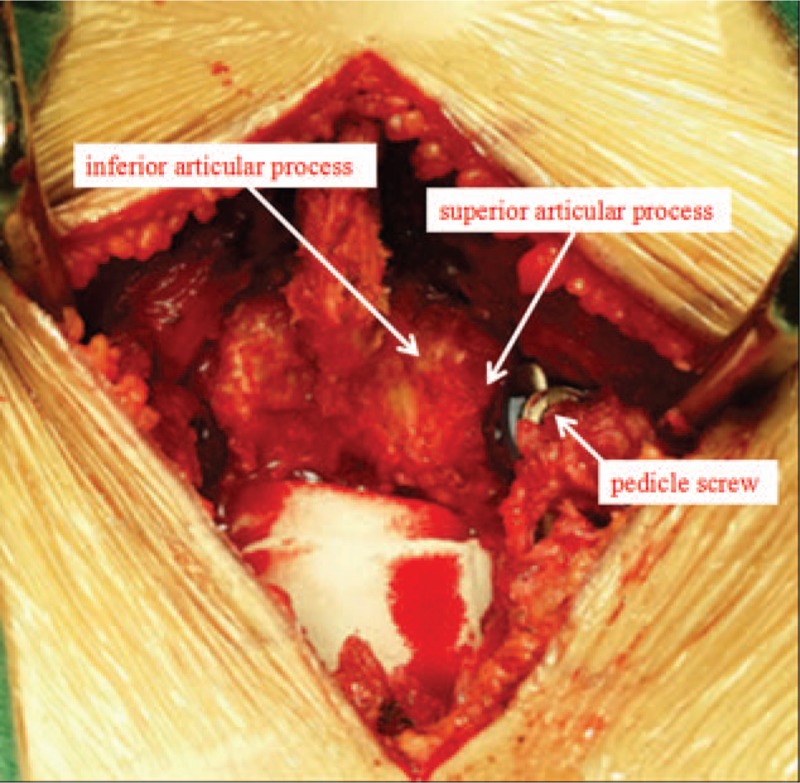
Intraoperative view of the complete reservation of facet joint, without superior facet joint violation.

The facet joints are a set of synovial, plane joints between the articular processes of 2 adjacent vertebrae, the biomechanical function of each pair of facet joints is to guide and limit movement of the spinal motion segment. In the lumbar spine, the facet joints function to protect the motion segment from anterior shear forces, excessive rotation and flexion, but have little influence on the range of lateral flexion. These functions can be disrupted by degeneration, dislocation, fracture, osteoarthritis, and surgery.^[34]^ In the procedure of pedicle screw insertion, the screw placement, which depends on the entry site selected, can damage the inferior facet of an adjacent segment.^[35]^ The transpedicular instrumentation technique must leave the facet joints adjacent to the top screw level intact. If not, an abnormal, alternate path of loading is established which makes the adjacent segment worse, and alterations in facet load-bearing capability from such an injury can potentially contribute to adjacent segment degeneration.^[36]^ Studies have demonstrated that pedicle screw insertion had effect on the articular facets of adjacent segments, which may be likely to lead to adjacent segment degeneration. Though several entry points have been described in the literature for the insertion of pedicle screws, the 2 which have been the most widely used are the intersection technique and the mamillary process technique.^[19]^ For experienced spinal surgeons, it is completely possible to prevent superior facet joint violation in the pedicle screw insertion (Fig. 6). While for new learners, it is inevitable to destroy the joint capsule and result in superior facet joint violation, especially for those who is not clear of the anatomical structures. Chung et al^[37]^ compared the 2 pedicle screw insertion techniques for facet joint violation in a cadaveric study, and reported that superior facet joint violation was more common with the use of the mamillary process technique as compared to the use of the intersection technique. Therefore, meticulous manipulation in exposure, identification of the facet joints accurately, adopt intersection technique may be of some help to prevent superior facet joint violation, and reduce the incidence of postoperative ASD.

There are several potential limitations in this study. First, the number of patients is relatively small, and the study may be under powered to detect the significance of some risk factors. Second, the study was conducted retrospectively by case selection, and was not randomized and controlled. Even with these issues in this study, we find that higher BMI, preoperative disc degeneration at adjacent level and intraoperative superior facet joint violation are risk factors for the occurrence of postoperative ASD. Patients who are overweight or obesity and with preoperative disc degeneration at adjacent segment should be fully informed the risk of ASD. For surgeons, it is essential to prevent superior facet joint violation in pedicle screw insertion procedure.
